# Active Video Games and Health Indicators in Children and Youth: A Systematic Review

**DOI:** 10.1371/journal.pone.0065351

**Published:** 2013-06-14

**Authors:** Allana G. LeBlanc, Jean-Philippe Chaput, Allison McFarlane, Rachel C. Colley, David Thivel, Stuart J. H. Biddle, Ralph Maddison, Scott T. Leatherdale, Mark S. Tremblay

**Affiliations:** 1 Healthy Active Living and Obesity Research Group, Children's Hospital of Eastern Ontario Research Institute, Ottawa, Ontario, Canada; 2 Department of Pediatrics, University of Ottawa, Ottawa, Ontario, Canada; 3 School of Sport, Exercise & Health Sciences, Loughborough University, Loughborough & The NIHR Leicester-Loughborough Diet, Lifestyle and Physical Activity Biomedical Research Unit, United Kingdom; 4 National Institute for Health Innovation, University of Auckland, Auckland, New Zealand; 5 School of Public Health and Health Systems, University of Waterloo, Waterloo, Ontario, Canada; CUNY, United States of America

## Abstract

**Background:**

Active video games (AVGs) have gained interest as a way to increase physical activity in children and youth. The effect of AVGs on acute energy expenditure (EE) has previously been reported; however, the influence of AVGs on other health-related lifestyle indicators remains unclear.

**Objective:**

This systematic review aimed to explain the relationship between AVGs and nine health and behavioural indicators in the pediatric population (aged 0–17 years).

**Data sources:**

Online databases (MEDLINE, EMBASE, psycINFO, SPORTDiscus and Cochrane Central Database) and personal libraries were searched and content experts were consulted for additional material.

**Data selection:**

Included articles were required to have a measure of AVG and at least one relevant health or behaviour indicator: EE (both habitual and acute), adherence and appeal (i.e., participation and enjoyment), opportunity cost (both time and financial considerations, and adverse events), adiposity, cardiometabolic health, energy intake, adaptation (effects of continued play), learning and rehabilitation, and video game evolution (i.e., sustainability of AVG technology).

**Results:**

51 unique studies, represented in 52 articles were included in the review. Data were available from 1992 participants, aged 3–17 years, from 8 countries, and published from 2006–2012. Overall, AVGs are associated with acute increases in EE, but effects on habitual physical activity are not clear. Further, AVGs show promise when used for learning and rehabilitation within special populations. Evidence related to other indicators was limited and inconclusive.

**Conclusions:**

Controlled studies show that AVGs acutely increase light- to moderate-intensity physical activity; however, the findings about if or how AVG lead to increases in habitual physical activity or decreases in sedentary behaviour are less clear. Although AVGs may elicit some health benefits in special populations, there is not sufficient evidence to recommend AVGs as a means of increasing daily physical activity.

## Introduction

The majority of children and youth around the world do not meet current physical activity guidelines and are considered to be inactive [1]. Self-reported measures of physical activity (PA) from the Global School-based Student Health Survey and the Health Behaviour in School-Aged Children Study (HBSC) show that 80% of 13–15 year olds do not participate in at least 60 minutes of moderate- to vigorous-intensity physical activity (MVPA) daily [2]. Further, it is now understood that children and youth spend a significant part of their day being sedentary. International data from the HBSC study show that 66% of girls and 68% of boys watch more than two hours of television per day [2], and data from a Canadian study show that youth accumulate an average of 7.8 hours of screen time daily [3].

High levels of habitual sedentary time (especially via screen-based activities) are associated with a range of negative health and behavioural indicators including poorer measures of body composition, fitness, self-esteem, self-worth, pro-social behaviour, and/or academic achievement [4]. Thus, population health researchers have started to develop novel interventions that use screen-based technology as part of the solution rather than part of the problem. One such intervention is the use of active video games (AVGs), or screen-based activities that require increased PA to play the game compared to conventional sedentary, or passive, video games (see Table 1).

**Table 1:** Definitions used to guide the systematic review.

**Passive video game**	An electronic or computerized game played seated by manipulating images on a video display or television screen, using a conventional gamepad controller (e.g. a conventional hand-held game).
**Active video game**	A video game that requires physical activity beyond that of a passive game (i.e. conventional hand-held games). Active video games rely on technology that tracks body movement or reaction for the game to progress.
**Traditional physical activity**	Any bodily movement produced by skeletal muscles that requires energy expenditure without the use of an electronic gaming system or display device.

AVGs have the potential to increase habitual PA and improve measures of cardiometabolic health among children and youth who would otherwise be spending time in sedentary, screen-based activities. Manipulating the gaming environment as an intervention tool for increasing PA is reinforced by recent findings showing that playing AVGs acutely increases EE compared to sedentary video games [5]–[10]. However, there is evidence to suggest that both children and adults may compensate for exercise interventions by decreasing spontaneous PA for the remainder of the day such that the net PA remains unaffected [11], [12]. Thus, from a public heath standpoint, it is important to examine the habitual and long-term impact of AVGs on a range of health and behaviour indicators to better appreciate the potential benefits (and potential risks) of AVGs. The objective of this systematic review is to present current evidence on the relationship between AVGs and several health and behavioural indicators in children and youth aged 0–17 years.

## Methods

### Quality Assessment

The GRADE (Grading of Recommendations Assessment, Development and Evaluation) framework was used to guide our review including *a-priori* ranking of health indicators and quality assessment of the evidence. Quality of evidence for each health indicator was assessed based on study design, risk of bias, consistency of results, directness of the intervention, precision of results, and possibility of a dose-response gradient. Details on data extraction are presented in the following sections. Details on GRADE methodology can be found elsewhere [13].

### Study Inclusion Criteria

To be included, studies needed to have a specific measure of time spent using AVGs using direct (e.g., accelerometer, pedometer or computer memory) or indirect (e.g., self- or parent-report) measurement, and a measure of at least one relevant health or behaviour indicator. Relevant health and behaviour indicators were chosen *a priori* by an expert panel (paper authors) and prioritized based on group consensus (Table 2).

**Table 2:** *A priori* consensus rankings assigned by the Guideline Development and Research Committee for each health indicator by age group.

Outcome	Priority
Physical Activity and Energy Expenditure	Critical
- Physical activity (light, moderate, vigorous intensity)	
- Sedentary behaviour (EE ≤1.5 METs and a sitting or reclining posture)	
- Activity compensation (i.e., is active video gaming replacing another activity, are children more/less likely to be physically active/sedentary as a result of playing active video games)	
Adherence and appeal	Critical
- Adherence to a program focused on an active video gaming vs. traditional physical activity	
- Adherence and appeal of active video games vs. passive games	
- Appeal (e.g., values and preferences for those who don't enjoy traditional physical activity)	
Opportunity cost	Critical
- Financial cost associated with traditional physical activity (e.g., equipment and registration of hockey, soccer etc.) vs. active video gaming (e.g., updating gaming console, games, accessories)	
- Time spent on active video games instead of traditional physical activity (i.e., does one replace the other)	
- Injury related to video game playing (e.g., injury due to over-exertion, accident, improper use)	
Adiposity	Important
- Body composition and measures of overweight or obesity (e.g., body mass index (BMI), waist circumference, skin-folds, bio-impedance analysis (BIA), dual-energy X-ray absorptiometry (DXA or DEXA))	
Cardiometabolic health indicators	Important
- Measures of cardiometablolic health (e.g., plasma lipids, lipoprotein concentrations (e.g. LDL-cholesterol, triglycerides), hypertension, fasting glucose, insulin resistance, inflammatory markers (e.g., C-reactive protein))	
Energy intake	Important
- Does EI increase/decrease when playing active video games	
- Differences in EI between passive and active video gamers	
Adaptation	Important
- Learned behaviour (i.e., tricks/tactics that change overall EE)	
- Controlled lab conditions vs. uncontrolled real life conditions (i.e., do EE, enjoyment, adherence differ?)	
Learning and rehabilitation	Important
- Rehabilitation (i.e., to help children with either chronic or acute conditions increase EE, movement acquisition or skills relevant to independence and tasks of daily living)	
- Effectiveness to teach new (or develop) fundamental movement skills (i.e., are active video games effective and feasible)	
Video game evolution	Important
- Do outcomes differ between types of active video games (e.g., is EE, EI or adherence different between consoles or gaming systems)	
- With respect to available technology (i.e. are active video games sustainable)	

**Note:** Health indicators were ranked based on whether they were critical for decision-making, important but not critical, or of low importance for decision-making. The focus when searching and summarizing the evidence was on indicators that were important or critical. Rankings were based on the GRADE framework [13].

### Study Exclusion Criteria

All published, peer-reviewed studies were eligible for inclusion; no date limits were imposed, but due to feasibility, studies in languages other than English or French were excluded. Studies were excluded if the mean age of participants was greater than 17.99 years; if the study examined only passive video games; if there was more than one aspect to the intervention that may have confounded the results (e.g., an intervention that included both AVG and diet components); or if the outcome of interest was not included in our list of relevant health and behavioural indicators.

### Search Strategy

The following electronic bibliographic databases were searched: MEDLINE, EMBASE, psycINFO, SPORTDiscus and Cochrane Central Database. The search strategy was created and run by AGL (see Appendix S1). Database searches were limited to studies involving children and youth aged 0–17 years. References were extracted from the OVID, EBSCO and Cochrane interfaces and imported into Reference Manager Software (Version 11, Thompson Reuters, San Francisco, CA).

Titles and abstracts of potentially relevant articles were screened by two independent reviewers (AM, and one of JPC, RCC, AGL, or DT), and full text copies were obtained for articles meeting initial screening criteria. Full text articles were screened in duplicate for inclusion in the review (AM and one of JPC, RCC, AGL or DT); any discrepancies were discussed, and resolved by the reviewers. In addition to our search, seven key content experts were contacted and asked to identify what they deemed important papers in the field.

### Data Extraction and Analysis

Data extraction was completed by one reviewer and checked by another for accuracy (one of JPC, AGL or AM). One reviewer (AGL) independently assessed the quality of evidence for all studies [13]. Reviewers were not blinded to the author names or journal titles when extracting data. Studies were divided by health or behavioural indicator (some studies examined more than one indicator) and by study design.

## Results

**Figure 1:**
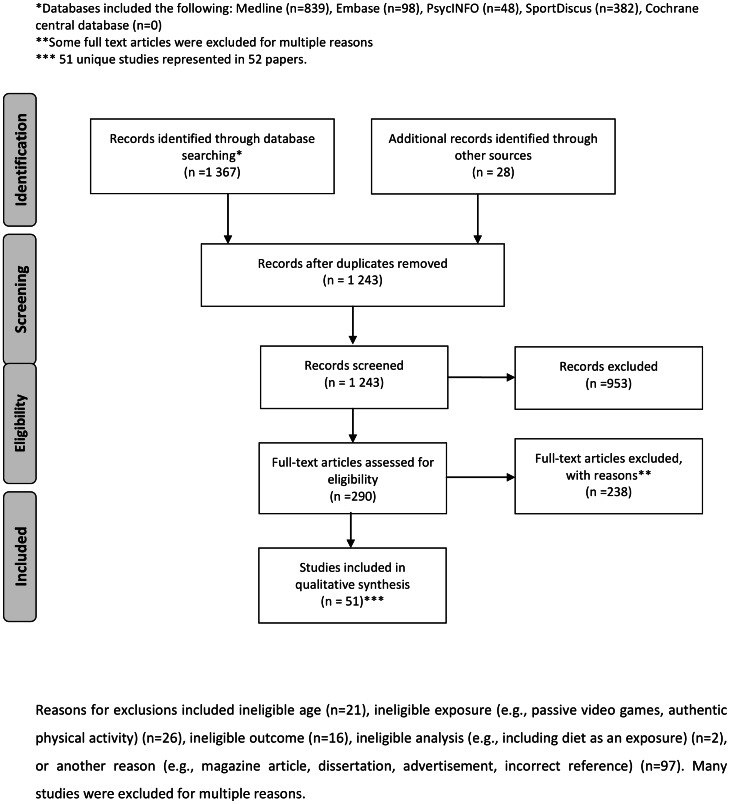
Prisma flow diagram of included studies.

Figure 1 shows the PRISMA flow diagram for study inclusion and exclusion. Table 3 provides a summary of all studies included in the review. Quality of evidence, by health or behaviour indicator, can be found in Tables 4–10. The indicators of interest represented in the included studies were energy expenditure (n = 35), adherence and appeal (n = 18), opportunity cost (n = 2), adiposity (n = 9), cardiometabolic health indicators (n = 3), energy intake (n = 2), and learning and rehabilitation (n = 9). No studies examining the relationship between AVG play and adaptation, or evolution of video games, were found. Many studies included results for more than one health indicator and were presented accordingly. Due to heterogeneity in AVGs used in the included studies (e.g., brand of gaming consoles, game type, playing time), a meta-analysis was not possible. Qualitative synthesis was conducted for all included studies.

**Table 3:** Descriptive characteristics of included studies.

First Author	Year	Country	Study Design	Population (*n*)	Age group	Intervention or exposure	Outcome and measure
Adamo [29]	2010	Canada	Randomized controlled trial	26Boys = 26Girls = 12	Age range = 12–17 yrsMean = 14.5 yrs	GameBike for Playstation (2, 60 min sessions/week, 10 weeks)	Energy expenditure (peak HR, average kcal expended, distance)Adiposity (BMI, % BF, waist circumference)Cardiometabolic health indicators (total cholesterol, HDL, LDL, total cholesterol/HDL ratio, fasting blood glucose, fasting insulin, TG)
Bailey [31]	2011	USA	Intervention	39Boys = 19Girls = 20	Age range = 9–13 yrsMean = 11.5 yrs	DDR, LightSpace, Wii, Cybex Trazer, Sportwall, Xavix (10–15 mins/day)	Energy expenditure [indirect calorimetry]Adherence and appeal (enjoyment [10-point Likert scale])Adiposity (BMI)
Baranowski [20]	2012	USA	Randomized controlled trial	78Boys = 40Girls = 38	Age range = 9–12 yrsMean = 11.3 yrs	Wii (7 weeks)	Energy expenditure (PA [accelerometry-min/day])Adherence and appeal (qualitative self-report)
Berg [59]	2012	USA	Case report	1	12 yrs	Wii (4, 20 min sessions/week, 8 weeks)	Learning and rehabilitation (Bruininks-Oseretsky Test of Motor Proficiency)
Bethea [25]	2012	USA	Intervention	28Boys = 18Girls = 10	Age range = 9–11 yrsMean = 9.9 yrs	DDR (3 days/week, 30 weeks)	Energy expenditure (physical activity [VO2max])Cardiometabolic (fasting metabolic profile [total cholesterol, LDL, HDL, TG, and glucose-mg/dL])Adiposity (BMI)
Chang [60]	2011	China	Case study	2Boys = 1Girls = 1	Age range = 16–17 yrsMean = 16.5 yrs	Kinerehab (2 sessions/day, 34 days), Microsoft Kinect	Learning and rehabilitation (number of correct movements)
Chin A Paw [49]	2008	Netherlands	Randomized controlled study	16Boys = 2Girls = 14	Age range = 9–12 yrsMean = 10.6 yrs	Interactive dance simulation video game (12 weeks)	Adherence and appeal [focus group discussions]Adiposity (aerobic fitness [shuttle run], BMI, PA [questionnaire]).
Deutsch [63]	2008	USA	Case study	1Boys = 1Girls = 0	Age = 13 yrs	Wii (11, 60–90 min sessions, 4 weeks)	Learning and rehabilitation (visual-perceptual processing [Test of Visual Perceptual Skills], postural control [weight distribution and sway measures], functional mobility [gait distance]).
Dixon [51]	2010	New Zealand	Cross sectional	37Boys = 22Girls = 15	Age range = 10–14 yrs	EyeToy, DDR (20–30 min/game)	Adherence and appeal (preference [focus group interviews])
Duncan [17]	2011	England	Randomized controlled trial	40Boys = 20Girls = 20	Age range = 10–11 yrsMean = 10.8 yrs	Gamercize (2 sessions/week, 6 weeks)	Energy expenditure (PA, HR [pedometry-steps/min], [% MVPA])
Duncan [16]	2010	England	Randomized controlled trial	30Boys = 12Girls = 18	Age range = 10–12 yrsMean = 10.4 yrs	Wii (2 sessions/week, 6 weeks)	Energy expenditure (PA, HR [pedometry-steps/min], [% MVPA])
Epstein [52]	2007	USA	Randomized controlled trial	35Boys = 18Girls = 17	Age range = 8–12 yrsMean = 10.8 yrs	DDR (2 min/6 games, 2 sessions)	Adherence and appeal (7-point Likert scale)
Errickson [22]	2012	USA	Randomized controlled trial	60Boys = 27Girls = 33	Age range = 8–12 yrsMean = 7.5 yrs	DDR (120 min/week, 10 weeks)	Energy expenditure (accelerometry, DDR logs, and Playstation2 memory cards)
Fawkner [32]	2010	Scotland	Cross sectional	19Boys = 0Girls = 19	Age range = 13–15 yrsMean = 14.0 yrs	Zigzag Xer-Dance (30 mins, 4 sessions, 6 weeks)	Energy expenditure (REE, EE, HR)
Fogel [27]	2010	USA	Cross sectional	4Boys = 2Girls = 2	Age range = 9–11 yrsMean = 9 yrs	DDR, Gamercize, Three Rivers Game Cycle, Dog Fighter, Cateye, Wii, iTech Fitness XrBoard, Fit Interactive 3 Kick (30 min/game)	Energy expenditure (PA)Adherence and appeal (preference rating)
Gao [28]	2011	USA	Cross sectional	280Boys = 156Girls = 124	Age range = 12–15 yrsMean = 13.59 yrs	DDR (1 min bouts, 9 sessions)	Energy expenditure (PA [accelerometry]-MVPA)Adherence and appeal (7-point Likert scale)
Getchell [64]	2012	USA	Case-control	30Boys = 18Girls = 12	Age range = 15–20 yrsMean = 17.5 yrs w Autism, 17.23 yrs without	Wii, DDR (20 min bouts, 2 weeks)	Learning and rehabilitation (MVPA, EE [accelerometry])
Graf [33]	2009	USA	Cross sectional	23Boys = 14Girls = 9	Age range = 10–13 yrsMean = 11.9 yrs	DDR, Wii (2, 30 min sessions, 4 weeks)	Energy expenditure (EE [indirect calorimetry-kJ/min], step rate [accelerometry-steps/min], RPE, HR [Borg Scale]
Graves [19]	2010	England	Randomized controlled trial	42Boys = 28Girls = 14	Age range = 8–10 yrsMean = 9.2 yrs	jOG (12 weeks)	Energy expenditure [accelerometry-counts/min]Adherence and appeal [behavior preference survey]
Graves [35]	2010	England	Cross sectional	14Boys = 10Girls = 4	Age range = 11–17 yrsMean = 15.8 yrs	Wii (1, 70 min session)	Energy expenditure (REE, RHR, VO2, EE, and HR)Adherence and appeal (modified Physical Activity Enjoyment Scale)
Graves [34]	2008	England	Cross sectional	13Boys = 7Girls = 6	Age range = 11–17 yrsMean = 15.1 yrs	Wii (1, 60 min session)	Energy expenditure (PA [accelerometry-J/kg/min])
Graves [14]	2008	England	Cross sectional	11Boys = 6Girls = 5	Age range = 13–15 yrsMean = 14.6 yrs	Wii (1, 45 min session)	Energy expenditure [indirect calorimetry-kJ/kg/min]
Jannink [57]	2008	Netherlands	Randomized controlled trial	10Boys = 9Girls = 1	Age range = 7–16 yrsMean = 11.75 yrs	EyeToy (6 weeks)	Learning and rehabilitation (user satisfaction [post exercise questionnaire], functional outcome [Melbourne Assessment scores])
Jones [53]	2009	USA	Cross sectional	21Boys = 8Girls = 13	Age range = birth – 30+ (separated by groups ie: birth-5, 6–10, 11–15 yrs)Mean = 16 yrs	Wii	Opportunity cost (injury [self-reported])
Lam [36]	2011	China	Cross sectional	79Boys = 40Girls = 39	Age range = 9–12 yrsMean = 10.85 yrs	XaviX (2, 60 min sessions)	Energy expenditure (PA [accelerometry], HR)
Lanningham-Foster [37]	2009	USA	Cross sectional	22Boys = 11Girls = 11	Age range = 10–14 yrsMean = 12.1 yrs	Wii (1, 10 min session)	Energy expenditure [indirect calorimetry-kcal/hr/kg]
Lanningham-Foster [38]	2006	USA	Cross sectional	25Boys = 12Girls = 13	Age range = 8–12 yrsMean = 9.7 yrs	EyeToy, DDR (2, 15 min sessions)	Energy expenditure [indirect calorimetry-kJ/hr/kg]
Maddison [55] *	2012	USA	Randomized controlled trial	322Boys = 160Girls = 162	Age range = 10–14 yrsMean = 11.6 yrs	EyeToy (3 months)	Energy expenditure (MVPA [accelerometry], VO2max)Adiposity (BMI-kg/m^2^)Energy intake (snack frequency)
Maddison [39]	2007	New Zealand	Cross sectional	21Boys = 11Girls = 10	Age range = 10–14 yrsMean = 12.4 yrs	EyeToy (1, 25–40 min session)	Energy expenditure (HR [indirect calorimetry]
Maddison [21] *	2011	New Zealand	Randomized controlled trial	322Boys = 160Girls = 162	Age range = 10–14 yrs	EyeToy (1, 25–40 min session, 3 months)	Energy expenditure (MVPA, VO_2_max [20-m shuttle run, accelerometry])Adiposity (BMI, % body fat)Energy Intake [self-reported food snacking]
Madsen [56]	2007	USA	Randomized controlled trial	30Boys = 12Girls = 18	Age range = 9–18 yrsMean = 13.0 yrs	DDR (6 months)	Adiposity (BMI)Adherence and appeal
Maloney [15]	2008	USA	Randomized controlled trial	60Boys = 30Girls = 30	Age range = 7–8 yrsMean = 7.5 yrs	DDR (4 sessions, 28 weeks)	Energy expenditure (MVPA [accelerometry-min/day])Adiposity (BMI)Adherence and appeal [satisfaction survey]
Maloney [23]	2012	USA	Randomized controlled trial	65Boys = 31Girls = 34	Age range = 9–17 yrsMean = 12.32 yrs	DDR (12 weeks)	Energy expenditure (PA [self-reported-frequency, pedometry-steps/day, accelerometry-min/day])
Mellecker [40]	2010	China	Intervention	27Boys = 10Girls = 17	Age range = 9–13 yrsMean = 11.0 yrs	X-BOX 360 on an adapted treadmill (4, 60 min sessions)	Energy intake [kcal intake]
Mellecker [41]	2008	China	Cross sectional	18Boys = 11Girls = 7	Age range = 6–12 yrsMean = 9.6 yrs	XaviX (10 min session)	Energy expenditure (REE, EE [indirect calorimetry- kcal/min], HR [beats/min])
Mitre [42]	2011	USA	Cross sectional	19Boys = 11Girls = 8	Age range = 8–12 yrsMean = 10 yrs	Wii (10 min bouts)	Energy expenditure [indirect calorimeter]Adiposity (BMI)
Murphy [24]	2009	USA	Randomized controlled trial	35Boys = 18Girls = 17	Age range = 7–12 yrsMean = 10.21 yrs	DDR (12 weeks)	Energy expenditure (HR, VO_2_peak [cycle ergometer])Cardiometabolic health indicators (HDL, LDL, TG, insulin, glucose)Adiposity (BMI)
Ni Mhurchu [18]	2008	New Zealand	Randomized controlled trial	20Boys = 12Girls = 8	Age range = 10.5–13.5 yrsMean = 12 yrs	EyeToy (12 weeks)	Energy expenditure [accelerometry, Physical Activity Questionnaire for Children, activity log]Adiposity (BMI, waist circumference)
Owens [26]	2011	USA	Intervention	12Boys = 6Girls = 6	Age range = 8–13 yrsMean = 10 yrs	Wii (12 weeks)	Energy expenditure (MVPA [accelerometry] muscular fitness [push-ups], aerobic fitness [VO_2_] and flexibility [trunk flexion])Adiposity (BMI, %BF)
Paez [50]	2009	USA	Randomized controlled trial	60Boys = 31Girls = 29	Age range = 7–8 yrsMean = 7.5 yrs	DDR (4, 120 min sessions/week, 10 weeks)	Energy expenditure (PA [accelerometry], BMI)Adherence and appeal [PA logs]
Penko [30]	2010	USA	Cross sectional	24Boys = 12Girls = 12	Age range = 8–12 yrsMean = 10.4 yrs	Wii (10 min bouts +)	Energy expenditure (HR [telemetry monitor], VO_2_ [indirect calorimetry])Adherence and appeal (Relative Reinforcing Value [RPE scale, likeability scale])
Perron [43]	2011	USA	Cross sectional	30Boys = 19Girls = 11	Age range = 7–12 yrsMean = 9.4 yrs	Wii, EA SPORTS Active (1, 20–25 min session)	Energy expenditure (RPE [OMNI scale], HR, PA [accelerometry])
Roemmich [47]	2012	USA	Cross sectional	44Boys = 22Girls = 22	Age range = 8–12 yrsMean = 10.15 yrs	Wii (1, 60 min session)	Energy Expenditure (PA/MVPA [accelerometry], HR)Adherence and appeal (likert scale likeability ratings, choice of video game)
Rubin [54]	2010	USA	Case report	4Boys = 3Girls = 1	Age range = 3–9 yrsMean = 6.75	Wii	Opportunity cost (injury [self-reported pain])
Shih [61]	2011	China	Intervention	2Boys = 1Girls = 1	Age range = 17–18 yrsMean = 17.5 yrs	Wii (4–6, 3 min sessions/day, 51–63 sessions)	Learning and rehabilitation (number of correct responses)
Shih [62]	2011	China	Intervention	4Boys = 3Girls = 1	Age range = 14–17 yrsMean = 15.25 yrs	Wii (4–6, 3 min sessions/day, 60–63 sessions)	Learning and rehabilitation (number of correct responses)
Sit [44]	2010	China	Cross sectional	70Boys = 35Girls = 35	Age range = 9–12 yrsMean = 10.87 yrs	XAviX , Aerostep (2, 60 min sessions)	Energy expenditure (HR, minute ventilation, VO_2_)Adherence and appeal (time spent on chosen game)
Smallwood [45]	2012	England	Cross sectional	18Boys = 10Girls = 8	Age range = 11–15 yrsMean = 13.4 yrs	Kinect	Energy expenditure (HR, VO_2_, EE (kcal/min))
Straker [46]	2007	Australia	Cross sectional	20Boys = 12Girls = 8	Age range = 9–12 yrs	EyeToy	Energy expenditure (HR, EE (kcal/min/kg) [indirect calorimetry], minute ventilation (L/min), O_2_ uptake (ml/min/kg))
White [48]	2010	New Zealand	Cross sectional	26Boys = 26Girls = 0	Age range = 10–13 yrsMean = 11.4 yrs	Wii (2 sessions)	Energy expenditure (RMR, EE, V0_2_ peak)
Widman [65]	2006	USA	Intervention	8Boys = 4Girls = 4	Age range = 15–19 yrsMean = 16.48 yrs	GameCycle (16 weeks)	Learning and rehabilitation (peak V0_2_ [arm ergometer] peak HR, max work output, aerobic endurance, RPE [Borg scale], user satisfaction [survey])
Wuang [58]	2010	China	Randomized control trial	115	Age range = 7–12 yrs	Wii (2, 60 min sessions/week, 24 weeks)	Learning and rehabilitation ([Bruininks–Oseretsky Test of Motor Proficiency, The Developmental Test of Visual Motor Integration, The Test of Sensory Integration Function])

* Maddison and Maddison used participant data from the same study but presented in two manuscripts.

AVG, active video games; BMI, body mass index; EE, energy expenditure, DDR, Dance Dance Revolution; HR, heart rate, HDL, high-density lipoprotein; LDL, low-density lipoprotein; MVPA, moderate- to vigorous-intensity physical activity; PA, physical activity, REE, resting energy expenditure; RMR, resting metabolic rate; RPE, rating of perceived exertion; TG, triglycerides.

**Table 4:** Association between active video games and energy expenditure in children and youth.

Quality assessment							No of participants	Absolute effect (confidence interval, standard error)	Quality	Importance
No of studies	Design	Risk of bias	Inconsistency	Indirectness	Imprecision	Other considerations				
**Habitual EE associated with AVG (age range between 7 and 17 years, follow-up between 6 weeks and 3 months, measured through self- and parent-report questionnaire, pedometry, accelerometer, fitness testing)**										
11	RCT	No serious risk of bias	Serious inconsistency^a^^, ^ b ^, ^ c ^, ^ d ^, ^ e	No serious indirectness	No serious imprecision	None	725^f^	146.40±37.86 min/day, 16.37±12.26 min/day^g^ baseline = 10.0±7.7 mpw, week 10 = 16.2±11.8 mpw, p<0.0005^h^	⊕⊕⊕Ο MODERATE	CRITICAL
								MD = 2.97±4.99, p = 0.013^i^		
								MD = −18.98, p = 0.003^j^		
								29.5±4.5 ml/kg/min, p<0.01^k^		
6	Observational study^l^	No serious risk of bias	No serious inconsistency^m^	No serious indirectness	No serious imprecision	None	296	F 1, 28 = 15.6, p = 0.0001^n^ 9.2 min^o^	⊕⊕ΟΟ LOW	CRITICAL
								F(2, 558) = 352.45, p<0.01^p^		
**Acute EE associated with AVG (age range between 6 and 15 years, activity session between 15–70 minutes, measured through kcal, HR, VO2, METs, pedometry, accelerometry)**										
2	RCT	No serious risk of bias^q^	No serious inconsistency^r^	No serious indirectness	No serious imprecision	None	50	11.7±3.1 ml/kg/min^s^	⊕⊕⊕Ο MODERATE	CRITICAL
18	Observational^t^	No serious risk of bias^u^	No serious inconsistency	No serious indirectness	No serious imprecision	None	493	16.7–28.1 kJ/min^v^ 3.63±0.58; 3.65±0.54; 4.14±0.71^w^ 3.3; 2.9; 3.0^x^	⊕⊕ΟΟ LOW	CRITICAL
								190.6±22.2; 202.5±31.5; 198.1±33.9^y^		
								182.1±41.3; 200.5±54.0; 267.2±115.8^z^		
								190.8±34.6; 236.8±36.4; 188.2±31.0; 348.1±44.7;		
								384.9±81.1; 697.7±89.9^aa^		
								141±20 bpm^bb^		
								108±40%, 172±68^cc^		
								5.14±1.71(329%)^dd^		
								6.5±1.7, 5.9±1.8, 4.9±1.3, 2.9±0.3, 3.6±1.1^ee^		
								0.01 kcal/kg/min (39%), 0.03 kcal/kg/min (98%),		
								0.12 kcal/kg/min (451%)^ff^		
								0.63±0.011, p<0.001 kcal/kg/min^gg^		
								3.05±0.93^hh^		
								144.0±8.0 bpm, 136.5±9.6 bpm^ii^		
								118bpm, 131bpm^jj^		
								F(1, 10) = 4.37, p<0.03; F(1, 40) = 20.73, p<0.001^kk^		
								38.9% MVPA, 0.13 kcal.kg/min; 52.9%MVPA,		
								0.18 kcal/kg/min^ll^		
								0.125 kcal/kg/min, p<0.001^mm^		
								63–190%, p≤0.001; 56–184%, p≤0.001^nn^		

**Habitual EE:** Randomized trials [14]–[24], Observational [25]–[28], [56].

a No significant difference in objectively measured time spent engaging in PA between children who were given inactive video games and those that were given active video games [19].

b No significant different in total physical activity 12 weeks post intervention between active gaming and sedentary gaming group [19].

c Intervention had no effect on time spent engaging in MVPA (measured by accelerometer) or level of physical fitness (measured through VO2max test) [21].

d Twelve week DDR intervention had no effect on fitness test results post-intervention (step test), physical activity (light, moderate, or vigorous intensity measured by accelerometer) or step counts (measured by pedometer) [23].

e No significant difference in overall physical activity or MVPA between intervention and control groups at the end of a 12 week active video game intervention [18].

f To meet eligibility criteria, participants had to be overweight or obese as per International Obesity Task Force criteria.\ [21], [55].

g Time spent in moderate and vigorous intensity PA respectively, compared to 112.1±36.7 min/day of moderate intensity activity and 12.7 min/day of vigorous intensity activity for control group. No significance given [22].

h Represents the main effect of heart rate to explain percentage of time spent in MVPA was significantly lower in the intervention group, compared to the control group, across the intervention period [17].

i Children in the active gaming intervention group had significantly fewer steps per day that those in the control group after 6 weeks [16].

j Increase in vigorous physical activity in DDR intervention group at week 10 [15].

k Peak VO_2_ in the exercise group following a 12 week DDR intervention was significantly higher than the control group (24.3±4.8 ml/kg/min) [24].

l Includes 5 intervention [24]–[27], [55]; however, quantitative data for change in EE during intervention not shown and therefore study will be removed from further EE analysis [56].

m Three month at home Wii Fit intervention had no effect on measures of peak VO_2_, balance, flexibility, muscular strength, or time spend engaging in PA [26].

n Mean difference in VO2max (ml/kg/min) at 30-wks compared to baseline [25].

o Mean minutes of physical activity per session (compared to 1.6 min in physical education class) [27].

p Students spend more time in MVPA in fitness class (40.46%) and football (37.09%) class than playing DDR (7.91%) [28].

q Participants included obese individuals or overweight individuals with at least one co-morbidity (i.e. BMI>95th% or BMI>85th% + elevated glucose, triglycerides, LDL cholesterol or decreased HDL cholesterol) [29].

r No significant difference in average time spent pedaling (min/session), EE (kcal/session), time spent in moderate intensity PA (60–79% peak HR), or average distance pedaled (km) between GameBike intervention group and music only control group. Music only control group spent significantly more time in vigorous intensity PA (80–100% peak HR), (24.9±20.0) than the Game bike intervention group (13.7±12.8), p = 0.05 [29].

s VO2 during Wii Boxing was significantly higher than at rest, while playing sedentary video games and during treadmill walking (at 1.5 mph) (P<0.05). Data was also presented for mean heart rate and RPE but not presented here [30].

**Acute EE:** Randomized trials [29], [30], Observational studies [31]–[48].

t Includes 7 intervention studies [30]–[36] and 12 cross sectional studies [37]–[48].

u Participants were chosen from their physical education class because they were inactive, overweight, had low fitness scores, and good behaviour and attendance [26]; very little information given on recruitment, attrition, group allocation [32]; participants were chosen from government subsidized elementary schools and therefore results may not be generalizable to the general public [36]; participants were only included if they already owned EyeToy games [39]; participants were recruited from a convenience sample via University wide emails to faculty and staff, may not be representative to general population [43]; subsample of participants to complete ergospirometery was chosen from main sample based on ‘ability to follow directions and complete measurements during main study’ - may bias towards fitter children [47].

v EE for active video games (Wii boxing, DDR thirteen, Cybex Trazer Goalie Wars, LightSpace Bug Invasion, Sportwall, Xavix J-mat) compared to 4.6 kJ/min at rest [30].

w EE (kcal/min) compared to rest (1.18±0.5 kcal/min) [31].

x Increase in EE (kcal/hr/kg) of DDR and Will boxing (in boys and girls respectively) above rest compared to 3.0 times increase in EE associated with walking at 5.7 km/hr [32].

y EE (kj/kg/min) for Wii bowling, Wii tennis and Wii boxing respectively compared to 125.5±13.7 for sedentary video games (XBOX360). For all games, EE was less than traditional version Graves [35].

z EE (kj/kg/min) for Wii bowling, Wii tennis and Wii boxing respectively compared to 115.8±18.3 for sedentary video games (XBOX360). Boxing was associated with greater EE than the other games (p<0.05). Data also presented (but not reported here) for VO2 (l/min) and HR (bpm) [33].

aa EE (kj/kg/min) for Wii yoga, Wii conditioning, Wii balance, Wii aerobics, treadmill walking and treadmill running respectively compared to 111.7±22.7 at rest and 113.526.3 for handheld gaming. Data also presented (but not reported here) for VO2 (l/min) and HR (bpm) [14].

bb Cardiovascular effort per hour of play (in beats per minute) compared to 104±17 bpm when playing a seating internet game (p<0.01). Results were not significant for girls or for bowling type games [36].

cc Percent increase in EE above rest compared to 138±40% for treadmill walking at 1.5mph and 22±12% for sedentary video games [37].

dd Increase in EE (kcal/hr/kg) above rest when playing Nintendo Wii Sports compared to playing sedentary video games (1.67±0.37(40%)), p<0.0001 [38].

ee EE (kcal/min) playing ‘knockout’, ‘homerun’, ‘dance UK’, ‘Groove’ and AntiGrav respectively compared to 1.6±0.2 kcal/min playing a sedentary games and 1.3±0.2 at rest. VO2, HR, METs and activity monitor counts also measured but not reported here [39].

ff Increased in EE (kcal/kg/min and percent increase) above rest for seated bowling, XaviX bowling, and XaviX J-Mat respectively [40].

gg EE while using a walking media station was significantly higher than rest and while playing a seated video game. Study reports similar results for V0_2_ and heart rate but not reported here [41].

hh Increase in EE (kcal/kg/min) above rest. This was higher in lean (3.50±0.71 kcal/kg/hr) than overweight/obese participants (2.42±0.85 kcal/kg/min) [42].

ii Mean heart rate during activities using EA SPORTS Active and Wii Fit respectively were significantly higher than baseline measures of 107.1±18.6 bpm (with EA SPORTS Active) and 109.2±16.9 bpm (with Wii Fit) [43].

jj Mean heart rate during Dance Central and Kinect Sports Boxing respectively. Heart rates were 53% and 70% higher than rest, respectively (p<0.001) and 34% and 48% higher than during sedentary video game play respectively (p<0.001) [45].

kk Rate of EE while playing each of the traditional games was greater than the corresponding exergame; significant main effect as average intensity was 107% greater when children had access to traditional indoor games (basketball, boxing, golf, hockey) versus same version of exergame (Wii) [47].

ll Percent of time children engaged in MVPA during 57.7min XaviX bowling session and 55.3 min Aerostep running session [46].

mm EE while playing with EyeToy interactive video game was significantly higher than when using a handheld game, a gamepad, a keyboard or a wheel. Heart rate, minute ventilation, and oxygen uptake were also significantly higher (results presented in manuscript but not here) [44].

nn Percent increase in EE when participating in Wii bowling, boxing, tennis, Wii fit, skiing and step compared to rest and to sedentary video games respectively. Treadmill running (8.7±1.2 km/hr) was associated with a significantly greater increase in EE from rest than all active video games (442%). Results were also given for VO2peak, METs and HR but not presented here [48].

**Table 5:** Association between active video games and adherence and appeal in children and youth.

Quality assessment							No of participants	Absolute effect (confidence interval, standard error)	Quality	Importance
No of studies	Design	Risk of bias	Inconsistency	Indirectness	Imprecision	Other considerations				
**Adherence (age range between 9 and 12 years, intervention between 6 and 12 weeks, adherence measured through times self-report logs and computer memory chips).**										
8	RCT	No serious risk of bias	No serious inconsistency^a^^, ^ b ^, ^ c	No serious indirectness	No serious imprecision	None	208	MD = 9.95, p = 0.01, MD = 9.96, p = 0.01^d^	⊕⊕⊕⊕ HIGH	CRITICAL
								MD = −14.02, p = 0.05, MD = −18.98, p = 0.003^e^		
								0.14 hr/d; 0.25 hr/d; 0.37 hr/d; 0.25 hr/d^f^		
								week 1 = 147±145 mpw, week 10 = 60±61 mpw^g^		
								MD = −52 min/day CI:−101, −2, p = 0.04^h^		
4	Observational study^i^^, ^ j	No serious risk of bias	No serious inconsistency^k^	No serious indirectness	No serious imprecision	None	158^l^	45%^m^	⊕⊕ΟΟ LOW	CRITICAL
**Appeal (age range between7 and 17 years, data collected over single session to 10 week intervention, appeal measured through qualitative measures, likert scales, and scores on the Physical Activity Enjoyment Scale**										
2	RCT	No serious risk of bias	No serious inconsistency^n^	No serious indirectness	No serious imprecision	None	138		⊕⊕⊕Ο MODERATE	CRITICAL
7	Observational study^o^^, ^ p ^, ^ q	No serious risk of bias^r^	No serious inconsistency	No serious indirectness	No serious imprecision	None	440	F(5, 30) = 19.68, p<0.001^s^ F(2, 558) = 3.70, p<0.05; F(2, 558) = 13.27, p<0.01^t^	⊕⊕ΟΟ LOW	CRITICAL
								78.6±15.0; 78.8±16.9; 84.3±15.1; 90.4±9.8; 65.5±17.1; 59.8±24.8^u^		
								F(1, 40) = 17.8, p<0.001; F(1, 40) = 10.81, p<0.002); F(1, 40) = 43.57, 8.5±1.8 cm^v^		
								p<0.001^w^		

**Adherence:** Randomized trials [15]–[18], [21], [35], [49], [50], observational studies [25], [46], [51].

a Playing time for both an interactive dance video game decreased in both the home and multiplayer groups over the 12 week intervention but the change didn't reach significance; qualitative reports suggest that kids had technical problems with the game and found that it became boring [49].

b Children in the intervention and control group both decreased their time spent playing sedentary games, but the children in the control group decreased this time more, but did not reach significance (29.39 min; 95% CI: 219.38, 0.59 min; P = 0.06) [21].

c Size of TV, absence or other (sedentary) video games, and participation by others (parents, siblings, friends) were not significant predictors for time spent playing DDR or engaging in PA after 10-week intervention [50].

d For the first week of the intervention, children in the active gaming group had more steps than children in the control group (52.9 steps/min compared to 46.5 steps/min). At the midpoint and end of study children in the intervention group had significantly fewer steps per day than during the first week) [16].

e For the first week of the intervention, children in the active gaming group had more steps than children in the control group (52.9 steps/min compared to 46.5 steps/min). At the midpoint and end of study children in the intervention group had significantly fewer steps per day than during the first week) [17].

f Adjusted change score between intervention and control groups showing a decrease in sedentary video games and an increase in active video games at 6 and 12 weeks; decrease in sedentary video game playing at 6 weeks (score increased at 12 weeks showing a detrimental effect of the intervention, data not reported); decrease in TV viewing at 12 weeks; increase in total video game playing at 6 and 12 weeks [34].

g Mean use (minutes per week) of DDR at week 1 (peak usage) and week 10. Usage never reached ‘prescribed’ level of 120 minutes per week [15].

h Mean difference in average time spent playing active games (compared to inactive games) between intervention and control groups. Children in the intervention group also tended to spend less total time playing video games, but this did not reach significance (MD = −44 min/day, CI: −92, 2, *p = *0.06 [18].

i Includes 2 intervention studies [23], [49] and one cross-sectional study [46].

j Only qualitative data available and not included in this table [51].

k On average, no significant difference in time spent playing interactive versus online bowling or running game; however, non-overweight children spent more time on both interactive bowling (p>0.05) and running (p>0.01) than overweight participants [46].

l Number of participants at 30 weeks reported here. Number of participants at baseline = 28 and at 12 weeks = 25 [24].

m Percentage of children who had ‘lost interest’ in DDR by 3 months [24].

**Appeal:** One randomized trials [20], observational studies [27], [28], [30], [31], [35], [47], [52].

n Children reported that they like AVG because they “didn't have to go outside” and “doing activities that you wouldn't normally be able to do”. Things they didn't like were related to specific games such as “computer competitor would scream things”, “I couldn't understand a character”, “didn't have anyone to play with” or “didn't like difficulty level”. No specific data reported [20].

o Includes 6 intervention studies [27], [28], [30], [31], [35], [52] and 1 cross sectional study [47].

p Specific data not presented in paper. Boys enjoyed Wii boxing, Xavix J-mat more than girls (p≤0.05). Those with higher BMI enjoyed Sportwall more than those with a lower BMI (p≤0.05) [30].

q Data not reported, but the students preferred Wii bowling, boxing and DDR. The teacher reported that the exergaming was beneficial to the students, that it resulted in more student engagement, and they listened to instructions [27].

r Participants were chosen from their physical education class because they were inactive, overweight, had low fitness scores, and good behaviour and attendance [27]; Subsample of participants to complete ergospirometery was chosen from main sample based on ‘ability to follow directions and complete measurements during main study’ − may bias towards fitter children [47].

s Children liked DDR or DDR+video controller more than dance+music or dance+video conditions [52].

t Students reported higher intrinsic motivation and identified regulation towards fitness class than DDR [28].

u Scores on the Physical Activity Enjoyment Scale (PACES) for Wii conditioning, Wii balance, Wii aerobics, treadmill walking and treadmill running respectively compared to 60.8±18.8 for handheld gaming. Scores were significantly higher for Wii balance, Will conditioning, and Will aerobics (p≤0.003) and Wii Fit (p = 0.029) compared to handheld games. Treadmill walking was significantly different from Wii balance, Wii aerobics (p≤0.05). Treadmill running was significantly different from Wii balance, Wii aerobics (p≤0.035) [35].

v Rating of ‘liking’ on a visual analog scale. Participants rated Wii Boxing significantly higher than sedentary video games or treadmill walking (p<0.05) [30].

w Children liked traditional mini indoor basketball more than the exergame version. Liked the exergame version of golf more than indoor mini golf. Children spent an average of 87% more time in free play given access to exergames than indoor traditional games [47].

**Table 6:** Association between active video games and opportunity cost in children and youth.

Quality assessment							No of participants	Absolute effect (confidence interval, standard error)	Quality	Importance
No of studies	Design	Risk of bias	Inconsistency	Indirectness	Imprecision	Other considerations				
**Adherence and appeal (age range between birth and 16 years, measured through injury statistics and adverse events.**										
3	Observational study^a^	Serious risk of bias^b^	No serious inconsistency^c^	No serious indirectness	No serious imprecision^d^	None	25^e^	7, 6, 4, 19, 1, 1^f^	⊕ΟΟΟ VERY LOW	CRITICAL

a Includes 1 intervention study [21], 1 case report [54], and 1 cross sectional study [53].

b All 4 participants were regular clients in the author's chiropractic clinic; they were all given a brief examination when they presented new symptoms and treated accordingly [54].

c Of 8 serious adverse events reported during the three month study, none were deemed related to the study intervention (EyeToy) [21].

d Participants were briefly examined when they presented with new symptoms, possible that new symptoms were not directly related to Wii, no quantitative data presented [54].

e Represents cases (i.e. number) of injury associated with active video games as reported in the National Electronic Injury Surveillance System (representing emergency room visits from across the U.S.). Of 21 cases, 13 were in those were from birth to 15 years and 8 in those aged 16–30+ years [53].

f Represents number of injuries from being hit or hitting another object during the game (33%); strains or sprains (29%); contusions or abrasions (19%); lacerations (19%); factures (5%); and concussions (5%) respectively [53].

**Table 7:** Association between active video games and adiposity in children and youth.

Quality assessment							No of participants	Absolute effect (confidence interval, standard error)	Quality	Importance
No of studies	Design	Risk of bias	Inconsistency	Indirectness	Imprecision	Other considerations				
**Adiposity (mean age between 8 and 17 years, intervention studies were between 10 weeks and 6 months long, adiposity measured through BMI, BMI ** ***z*** **-score, % body fat, weight gain)**										
7	RCT^a^	No serious risk of bias	No serious inconsistency^b^	No serious indirectness	No serious imprecision	None	508^c^	42.111.7 %BF, 43.5±7.8 %BF^d^−0.24 CI: −0.44, −0.04, p = 0.02^e^−0.30(CI: −0.56, −0.04) p<0.0001; −0.41(CI: −1.19, 0.36) p<0.0001^f^0.91±1.54 lbs, 2.43±1.80 lbs, p = 0.017^g^MD = −1.4 cm CI:−2.68, −0.04, p = 0.04^h^	⊕⊕⊕⊕ HIGH	IMPORTANT
3	Observational study^i^	No serious risk of bias	No serious inconsistency^j^^, ^ k ^, ^ l	No serious indirectness	No serious imprecision	None	53^m^		⊕⊕ΟΟ LOW	IMPORTANT

Randomized trials [15], [18], [20], [22], [23], [28], [55], observational studies [24], [25], [56].

a Maddison [20] and Maddison [55] used data from the same study sample; results will be presented separately but number of participants is only counted once.

b There was no significant difference between change in BMI in intervention and control group after a 10-week DDR intervention [15]; the intervention had no significant effect on participant's weight over a 12 week DDR intervention [23].

c Participants included obese individuals or overweight individuals with at least one co-morbidity (i.e. BMI>95th% or BMI>85th% + elevated glucose, triglycerides, LDL cholesterol or decreased HDL cholesterol) [28]; to meet eligibility criteria, participants had to be overweight or obese as per International Obesity Task Force criteria [20], [54]; participants were required to be above the 85^th^ percentile for BMI [24].

d Post-intervention % body fat in music and GameBike group, respectively. GameBike group had a larger decrease in body fat (%) than group that exercised to music alone (pre-intervention body fat % = 45.2±9.6 and 43.7±11.8, respectively) [29].

e At 24 weeks, active gaming intervention group had significant decreases in BMI and zBMI (−0.06 CI: −0.12, 0.00; P = 0.04) [20].

f Difference in BMI and %body fat respectively between intervention and control group when controlled for aerobic fitness level [55].

g Weight gain in intervention and control groups after 12 week DDR physical activity intervention [28].

h Mean difference in waist circumference from baseline to end of 12 week active gaming intervention between intervention and control groups [18].

i Includes 3 intervention studies [25], [26], [56].

j No significant effect of 30-wk DDR intervention on BMI [26].

k When adjusted for baseline BMI z-score, DDR ise was not associated with change in BMI from baseline at either 3 or 6 months [56].

l Three month at home Wii Fit intervention had no effect on measures of body fat % or BMI [25].

m Number of participants at 30 weeks included here (number of participants at baseline = 28 and at 12 weeks = 25) [26]; to be included, participants had to be above the 95 percentile and recruited through a pediatric obesity clinic [56].

**Table 8:** Association between active video games and cardiometabolic health indicators in children and youth.

Quality assessment							No of participants	Absolute effect (confidence interval, standard error)	Quality	Importance
No of studies	Design	Risk of bias	Inconsistency	Indirectness	Imprecision	Other considerations				
**Cardiometabolic health indicators (age range between 7 and 17 years, intervention 10–30 weeks long, cardiometabolic health indicators measured through blood pressure (DBP, SBP), resting heart rate, cholesterol (HDL, LDL, total CHL, triglycerides), measures of insulin sensitivity (e.g. fasting insulin, HOMA)**										
2	RCT	No serious risk of bias	Serious inconsistency	No serious indirectness	No serious imprecision	None	61^a^	3.8±0.6 mmol/L, 4.1±0.9 mmol/L^b^84.4±7.3 mmHg, p>0.05^c^	⊕⊕⊕Ο MODERATE	IMPORTANT
1	Observational study^d^	No serious risk of bias	No serious inconsistency^e^	No serious indirectness	No serious imprecision	None	23^f^		⊕⊕ΟΟ LOW	IMPORTANT

Randomized trials [24], [29], observational studies [25].

a Participants included obese individuals or overweight individuals with at least one co-morbidity (i.e. BMI>95th% or BMI>85th% + elevated glucose, triglycerides, LDL cholesterol or decreased HDL cholesterol) [29].

b Post-intervention total cholesterol (mmol/L) in music and GameBike group, respectively. GameBike group had a larger decrease in total cholesterol than group that exercised to music alone (pre-intervention total cholesterol = 4.0±0.7 and 4.5±0.7, respectively). No difference on HDL, LDL, total cholesterol to HDL ration, fasting blood glucose, fasting insulin or triglycerides [29].

c Significant decrease in mean arterial pressure in exercise group (no change in control group). However, there was no significant difference between blood pressure (DBP, SBP), resting heart rate, cholesterol (HDL, LDL, total CHL, triglycerides) or measures of insulin sensitivity (insulin, HOMA) between intervention and control groups after a 12 week DDR intervention [24].

d Includes 1 prospective cohort study [25].

e No effect of 30-week DDR intervention on measures of blood pressure, fasting glucose, total, HDL, or LDL cholesterol or triglycerides post intervention [25].

f Number of participants at 30 weeks reported here. Number of participants at baseline = 28 and at 12 weeks = 25 [25].

**Table 9:** Association between active video games and energy intake in children and youth.

Quality assessment							No of participants	Absolute effect (confidence interval, standard error)	Quality	Importance
No of studies	Design	Risk of bias	Inconsistency	Indirectness	Imprecision	Other considerations				
**Energy intake (age range between 9 and 14 years, intervention between 6 sessions and 3 months follow-up, energy intake measured through snacking frequency and kcal intake)**										
1	RCT	No serious risk of bias	No serious inconsistency^a^	No serious indirectness	No serious imprecision	None	322		⊕⊕⊕⊕ HIGH	IMPORTANT
1	Observational	No serious risk of bias	No serious inconsistency^b^	No serious indirectness	No serious imprecision	None	27		⊕⊕ΟΟ LOW	IMPORTANT

Randomized trial [21], observational study [41].

a Average self-reported daily total energy consumed from snack food decreased in the active video game intervention group (compared to the sedentary video game control group), but change was not significant [21].

b No significant difference in energy intake between active gaming session (383±266 kcal/hr) versus seated gaming session (374±192 kcal/hr) [41].

**Table 10:** Association between active video games and learning and rehabilitation in children and youth.

Quality assessment							No of participants	Absolute effect (confidence interval, standard error)	Quality	Importance
No of studies	Design	Risk of bias	Inconsistency	Indirectness	Imprecision	Other considerations				
**Learning and rehabilitation (age range between 15 and 17 years, intervention between 2–16 weeks, learning and rehabilitation measured through Melbourne Assessment if Unilateral Upper Limb Function, correct movements, functional mobility, work capacity)**										
2	RCT	No serious risk of bias	No serious inconsistency	No serious indirectness	No serious imprecision	None	120^a^^, ^ b	0–15%^c^ F[34,272] = 42.31, p = 0.000^d^	⊕⊕⊕⊕ HIGH	IMPORTANT
7	Observational study^e^	Serious risk of bias^f^	No serious inconsistency	No serious indirectness	No serious imprecision	None	33^g^	2, -3^h^ p<0.05^i^	⊕ΟΟΟ VERY LOW	IMPORTANT
								41.1m^j^		
								98.5; 93.8; 76.4; 32.6; 25.9^k^		
								P<0.01^l^		
								p<0.01^m^		
								Pre: 65.5±9.7 W, post 77.7±7.1 W (P<0.015)^n^		

Randomized trials [57], [58], observational studies [59]–[65].

a Includes 5 children in the control group (continuing normal physiotherapy for cerebral palsy) and 5 in the intervention group [57].

b Includes children with diagnosed down syndrome but no other serious disease (n = 110) [58].

c Percent change of Melbourne Assessment if Unilateral Upper Limb Function in the intervention group compared to −1–4% change in the control group (p-value not reported) [57].

d Children in the intervention (Wii) group performed significantly better on all follow-up analyses than the control group. BOT-2 Bruininks–Oseretsky Test of Motor Proficiency-Second Edition; VMI, Developmental Test of Visual Motor Integration; TSIF, Test of Sensory Integration Function [58].

e Includes 4 intervention studies [59], [60], [63], [64], and 3 case studies [61], [62], [65].

f Case study of 1 participant diagnosed with Down Syndrome. Recruitment procedures were explained but difficult to generalize findings [59]; Inclusion was based on “readiness to participate” assessment by occupational therapist [60]; case study of 1 participant diagnosed with spastic diplegic cerebral palsy [63]; no information on recruitment, participant characteristics or group assignment given [61]; no information on recruitment, participant characteristics or group assignment given [62].

g Study was case control (15 participants with autism spectrum disorder, 15 apparently healthy). Only data from case (i.e. autism group) is presented here [64].

h Change in BOT-2 composite score after 8 week intervention for manual and body coordination respectively. Both changes exceed the minimum detectable change that would be statistically significant when comparing different samples and the minimum important difference that represents a clinically significant difference [59].

i Both participants significantly increased correct movements when playing Kinect during the intervention [60].

j Increase in functional mobility (with forearm crutches) after training. This continued to increase post-intervention [61].

k Percent time spent in MVPA (during a 30 minute exercise bout) while walking, running, playing DDR, playing Wii Fit and playing Wii Sport respectively [64].

l Both groups significantly increased the number of correct answers they provided after the intervention (using Wii balance board to follow instructions) [61].

m Both participants increased the number of correct responses during the two intervention periods (first intervention = 51 weeks, second intervention = 63 [62].

n Change in arm crank maximum work capacity (W) pre and post intervention using GameCycle [65].

## Data Synthesis

### Physical Activity and Energy Expenditure

Studies were grouped depending on if they examined habitual activity (i.e., if AVG was associated with increased PA, decreased sedentary behaviour, or change in fitness), or acute EE (i.e., measured EE during a single bout of AVG play) (Table 4).

### Changes in habitual physical activity

Eleven randomized controlled trials (RCTs), and five observational studies examined the relationship between AVG play and habitual PA. The majority of the `RCTs reported that an AVG intervention had no effect on time spent engaging in total PA [14]–[19], MVPA [15], [18], [20], [21], or physical fitness (estimated via shuttle run test) [21]. Maloney et al. [15] suggested that a Dance Dance Revolution (DDR) intervention increased self-reported levels of PA (measured via self-report) but not objectively measured PA (measured via accelerometer). Baranowski et al. [20] found no difference in objectively measured PA between children who were given a passive video game or those who were given AVGs. Ni Mhurchu et al. [18] showed increased PA at 6 weeks but not at 12 weeks in those who received an AVG intervention compared to those who continued playing passive games. The remaining RCTs suggest AVGs do have an effect on habitual EE [22]–[24]. Errickson et al. [22] reported increases in weekly MVPA in the intervention group after a 10-week DDR intervention but statistical significance was not reported; Maloney et al. [23] reported increased vigorous PA (hours/week) after a 10-week DDR intervention; and Murphy et al. [24] reported increases in aerobic fitness (peak VO_2_) after a 12-week DDR intervention.

The observational studies provided inconsistent results. Bethea et al. [25] reported increased aerobic fitness (VO_2_max) after a 30-week DDR intervention; however, Owens et al. [26] reported no change in either aerobic or muscular fitness after three months of Wii Fit use. Finally, there were inconclusive results comparing AVGs to traditional PA. Fogel et al. [27] reported higher levels of PA when playing AVGs compared to physical education class; whereas Gao et al. [27] reported that students spent more time in MVPA during fitness class and playing football, than when playing DDR.

### Changes in acute energy expenditure

Two RCTs examined the effect of AVG on acute EE. The first reported no significant difference in average time spent pedaling (min/session), EE (kcal/session), time spent in MVPA (60–79% peak heart rate/session), or average distance pedaled (km/session) between a GameBike intervention group and music only exercise group [29]. However, the second study reported higher measures of oxygen consumption (VO_2_), heart rate, and rating of perceived exertion while playing Wii boxing than when compared to rest or light treadmill walking (1.5 mph) [30].

Seven intervention studies and 12 cross-sectional studies examined the EE of AVGs compared to rest or to sedentary video games and all reported significant increases in EE [31]–[48]. Three of these studies suggested that although AVGs increased EE above rest, and while playing sedentary video games, EE is still less than when participating in traditional PA [34], [47], [48].

### Adherence and appeal

Studies were grouped depending on if they examined adherence to playing AVGs (i.e., children continued to use AVG in the long term, or if it dropped off quickly), or appeal of AVG (i.e., if children and/or their parents enjoyed AVGs) (Table 5).

### Adherence to active video games

Of the eight RCTs that assessed adherence to AVG play, four reported high levels of adherence at the midpoint of the study, but significantly lower levels by the end of the interventions (interventions ranged from 10–12 weeks) [15]–[17], [35]. One study reported that adherence was lower at the end of the study but the difference did not reach statistical significance [49]. Paez et al. [50] were unable to determine any significant predictors of time spent playing DDR at the end of a 10-week intervention. Finally, two studies reported that although both groups played fewer sedentary video games by the end of the study, there was a trend towards less sedentary play in the AVG intervention group compared to the control group [18], [21].

Two intervention studies and one cross-sectional study reported on adherence to AVG play. Bethea et al. [24] reported that children decreased time spent playing AVGs by the end of the study. Sit et al. [44] reported that although there was no significant difference in time spent playing interactive versus online bowling or running game, normal-weight children spent more time on both interactive bowling (p<0.05) and running (p<0.01) than overweight participants. Finally, Dixon et al. [51] provided qualitative data saying that overall, both parents and children supported the idea of AVGs, but not at the expense of traditional PA.

### Appeal of active video games

One RCT presented qualitative data reporting that in general, children like AVGs, and things they did not like were game-specific [20]. Of the six intervention and one cross-sectional studies, the majority reported that in general, children and youth enjoyed AVGs [27], [28], [31], [35], [47]. Children enjoyed Wii Balance, Wii Aerobics and Wii Boxing more than treadmill walking or running [30], [34], and Wii Golf more than traditional mini golf [47]; however, they enjoyed indoor mini basketball more than the video game version [46] and showed higher intrinsic motivation to fitness class than to DDR [28]. They also enjoyed DDR (even when using a handheld controller) more than dancing with music or an instructional video [52]. Bailey et al. [31] reported that boys enjoyed Wii Boxing and Xavix J-mat more than girls. Finally, Roemmich et al. [47] reported that children spent an average of 87% more time in free play when given access to AVGs compared to indoor versions of traditional PA.

### Opportunity cost

This review identified one RCT and two observational studies reporting on adverse events associated with AVGs. The RCT reported that none of the adverse events that occurred during the study period were related to the AVG intervention (EyeToy) [21]. Two observational studies reported some injuries associated with AVG use (e.g., back pain, fractures, bruises) [53], [54]. No studies reported on the financial opportunity cost (e.g., spending money on AVGs instead of on more traditional PA such as sports equipment or swimming lessons) or behavioural opportunity cost (e.g., AVGs displacing traditional PA) (Table 6).

### Adiposity

Six RCTs (from seven papers), and three intervention studies were included. Results of the RCTs seemed to depend on weight status of the participants included in the study. Three of the RCTs included only overweight or obese participants and reported that AVGs helped to attenuate weight gain [21], [29], [55]; however, of the three RCTs that included normal-weight participants, only one reported attenuated weight gain in the intervention group [18]. The three intervention studies reported that AVG had no effect on attenuating weight gain or promoting weight loss in normal weight [25], [26] or overweight [56] participants (Table 7).

### Cardiometabolic health indicators

Two RCTs and one prospective cohort study reported on the relationship between AVGs and cardiometabolic health. After a 12-week DDR intervention with overweight children, Murphy et al. [24] reported a significant decrease in mean arterial pressure in the exercise intervention group, but no changes in blood pressure, high-density lipoprotein cholesterol, low-density lipoprotein cholesterol, total cholesterol, or measures of insulin sensitivity (HOMA, fasting insulin); however, Adamo et al. [29] reported a decrease in total cholesterol after a 10-week GameBike intervention in obese children but no changes in high-density lipoprotein cholesterol, low-density lipoprotein cholesterol, fasting blood glucose, fasting insulin or triglycerides (they did not report on mean arterial blood pressure). Finally, a 30-week prospective cohort study found no effect of DDR use on blood pressure, fasting glucose, total cholesterol, high-density lipoprotein cholesterol, low-density lipoprotein cholesterol, or triglycerides post intervention [24] (Table 8).

### Energy intake

Two studies examined energy intake during AVG play [21], [41]. One RCT reported that over a 24-week AVG intervention (EyeToy), average self-reported daily total energy consumed from snack food decreased in the intervention group (567±684 kcal/day) compared to the passive video game control group (708±948 kcal/day), but the change was not statistically significant [21]. The other study (4 sessions using X-box 360 on an adapted treadmill) reported no significant difference in energy intake between the AVG session (383±266 kcal/h) versus the seated gaming session (374±192 kcal/h) [41] (Table 9).

### Learning and rehabilitation

This review included two RCTs and seven observational studies examining the relationship between AVG and learning and rehabilitation. The first RCT used Nintendo Wii along with standard physiotherapy to treat those with cerebral palsy (compared to standard physiotherapy alone), and saw significant improvements in upper limb function [57]. The second RCT also reported significant improvements in motor proficiency after a Wii intervention in those with Down's syndrome [58]. All seven observational studies showed improvements in learning and rehabilitation after an AVG intervention (using Nintendo Wii, DDR or Microsoft Kinect). This included improvements in manual and body coordination [59], following movement cues and directions [60]–[62], functional mobility [63], and length of time spent at higher intensity of PA [64], [65] (Table 10).

## Interpretation

This systematic review is the first to provide a comprehensive understanding of the influence of AVGs on multiple health and behavioural indicators in children and youth. Existing evidence suggests that AVGs are able to increase EE above rest and when compared to playing passive video games. The studies included in the systematic review also showed that AVGs do not make a significant contribution to enable children and youth to meet guidelines of 60 minutes of moderate- to vigorous-intensity physical activity on a daily basis [1]; however, AVGs may increase light- to moderate-intensity physical activity at the expense of some sedentary behaviours (including sedentary video games). The appeal of AVGs is high for some children, but there is a lack of evidence suggesting long-term adherence. In overweight and obese children and youth, AVGs may attenuate weight gain whereas evidence in normal-weight children is inconclusive. Evidence for energy intake and AVG play is also inconclusive as is the effect of AVG interventions on cardiometabolic health indicators or opportunity cost. Finally, there is evidence to suggest that AVGs can be beneficial to improve motor skill proficiency and movement cues in populations with movement difficulties.

### Strengths and limitations

The main strength of this study is the use of high, international standards of developing and conducting a systematic review. As many decisions as possible were made *a priori* which helps to limit potential bias throughout the review. Furthermore, all steps of the review (i.e., inclusion criteria, exclusion criteria, data extraction, GRADE tables) were done in duplicate to minimize error. Further, the systematic review has been completed as per PRISMA guidelines (Appendix S2). Finally, we focused on many health and behavioural indicators (i.e., not just EE) with the hope of providing a thorough understanding of the relationship between AVGs and health in children and youth.

The main limitation to our study, and an area for future research, relates to the relatively low quality of studies in this field of research. Most studies included in this review had small sample sizes and short intervention periods, making it difficult to elucidate the true effects of these technologies on health and behavioural outcomes. Further, since many studies were underpowered, some results were not statistically significant (and therefore not reported here) but showed a trend towards significance. Future work should aim to use larger sample sizes to avoid being underpowered, and focus on using both direct (e.g., accelerometer, pedometer, heart rate) and indirect (e.g., self-, parent-, caregiver-report) measures to assess total AVG use. Both measures are needed to reflect the nuances associated with capturing AVG play such as body position or intensity of play. Moreover, multiple follow-up measurements over longer time periods are required so the longitudinal effects of AVG use can be better understood. It is also important that future work aims to harmonize methods for data collection and analysis so that meta-analyses can be performed. Moreover, the review included studies that were largely based on what could be deemed “first generation” AVGs, as such there will be a need to re-evaluate the evidence in the future as AVGs evolve (and the quality of the research designs improve).

### Future directions

Other reviews in this area have shown similar results to ours in that some AVGs are able to acutely increase light- to moderate-intensity PA in some children and youth but unable to elicit PA of high enough intensity, or volume to enable children to meet physical activity guidelines [5]–[10], [66]. More high quality, robustly designed and well powered studies are needed comparing AVGs to traditional PA (not just to rest or other sedentary games); comparing different types of video game consoles; measuring energy intake while playing AVGs (compared to a variety of both active and sedentary behaviours); assessing AVG use in limited areas that may be unsafe; assessing the ability of AVGs to displace sedentary time; examining the opportunity cost of AVGs (i.e., both time and financial considerations); and assessing behavioural compensation throughout the entire day.

### Conclusion

While controlled laboratory studies clearly demonstrate that a motivated player can obtain some light- to moderate-intensity PA from most AVGs, the findings are inconsistent about whether, or the circumstances under which, having an AVG results in sustained PA behaviour change, or for how long the behaviour change persists. Some of these games offer nuances on game play that could be related to increased PA or decreased sedentary behaviour. AVG technology is innovating at a rate that outpaces the related research. Higher quality research is needed that tests conceptual models of how different AVGs may relate to the initiation and maintenance of increased PA or decreased sedentary behaviour and understand their effects on health outcomes to resolve these inconsistencies.

## Supporting Information

Appendix S1 **Search strategy.**
(DOC)

Appendix S2 **PRISMA presubmission checklist.**
(DOC)
