# Supplementary material for: Active Video Games and Health Indicators in Children and Youth: A Systematic Review
Source: PLoS One. 2013 Jun 14;8(6):e65351. doi: 10.1371/journal.pone.0065351 (PMC3683002; doi:10.1371/journal.pone.0065351)
Supplement: Appendix S1 — Search strategy. (DOC) [file pone.0065351.s001.doc]

**Appendix A**

Search Strategy

| **Medline** | **embase** | **psycINFO** | **Cochrane** | **Sport Discus** |
| --- | --- | --- | --- | --- |
| 1. (dance dance revolution or (dance simulation video or dance gaming)).tw. or exergame.mp. or active gam*.mp. or active video.mp. or wii.mp. or kinect.mp. or new generation computer.mp. | 1. dance dance revolution.tw. | 1. dance dance revolution.tw. | 1. dance dance revolution.tw. | dance dance revolution OR exergame OR active gam* OR active video OR wii OR kinect AND ( pediatric or paediatric or child* or newborn* or adolescen* or infan* or neonat* or youth* or teen* or baby* or babies* or preschool* or pre-school* or elementary school or elementary student*or kindergarten or nursery school* or grade school* or public school* or highschool* or high school* or schoolchild* or school child* ) |
| 2. video games/ | 2. (dance simulation video or dance gaming).tw. | 2. (dance simulation video or dance gaming).tw. | 2. (dance simulation video or dance gaming).tw. |  |
| 3. 1 or 2 | 3. exergame.mp. | 3. exergame.mp. | 3. exergame.mp. [mp=title, short title, abstract, full text, keywords, caption text] |  |
| 4. 3 and (child$ or adolescent or infan$).mp. | 4. active gam*.mp. | 4. active gam*.mp. | 4. active gam*.mp. [mp=title, short title, abstract, full text, keywords, caption text] |  |
|  | 5. new generation computer.mp. | 5. wii.mp. | 5. active gam*.mp. [mp=title, short title, abstract, full text, keywords, caption text] |  |
|  | 6. wii.mp. | 6. kinect.mp. | 6. video game.mp. [mp=title, short title, abstract, full text, keywords, caption text] |  |
|  | 7. kinect.mp. | 7. 1 or 2 or 3 or 4 or 5 or 6 | 7. wii.mp. [mp=title, short title, abstract, full text, keywords, caption text] |  |
|  | 8. 1 or 2 or 3 or 4 or 5 or 6 or 7 | 8. Computer Games/ | 8. kinect.mp. [mp=title, short title, abstract, full text, keywords, caption text] |  |
|  | 9. 8 and (child$ or adolescent or infan$).mp. | 9. 7 or 8 | 9. new generation computer.mp. [mp=title, short title, abstract, full text, keywords, caption text |  |
|  |  | 10. 9 and (child$ or adolescent or infan$).mp. |  |  |
|  |  | 11. 7 and (child$ or adolescent or infan$).mp |  |  |
